# Snake Venom Proteomics of Samar Cobra (Naja samarensis) from the Southern Philippines: Short Alpha-Neurotoxins as the Dominant Lethal Component Weakly Cross-Neutralized by the Philippine Cobra Antivenom

**DOI:** 10.3389/fphar.2021.727756

**Published:** 2021-12-24

**Authors:** Praneetha Palasuberniam, Yi Wei Chan, Kae Yi Tan, Choo Hock Tan

**Affiliations:** ^1^ Venom Research and Toxicology Laboratory, Department of Pharmacology, Faculty of Medicine, University of Malaya, Kuala Lumpur, Malaysia; ^2^ Department of Biomedical Sciences, Faculty of Medicine and Health Sciences, University Malaysia Sabah, Kota Kinabalu, Malaysia; ^3^ Protein and Interactomics Laboratory, Department of Molecular Medicine, Faculty of Medicine, University of Malaya, Kuala Lumpur, Malaysia

**Keywords:** Southern Philippine Cobra, spitting cobra, venomics, alpha-neurotoxin, immunoreactivity

## Abstract

The Samar Cobra, *Naja samarensis*, is endemic to the southern Philippines and is a WHO-listed Category 1 venomous snake species of medical importance. Envenomation caused by *N. samarensis* results in neurotoxicity, while there is no species-specific antivenom available for its treatment. The composition and neutralization of *N. samarensis* venom remain largely unknown to date. This study thus aimed to investigate the venom proteome of *N. samarensis* for a comprehensive profiling of the venom composition, and to examine the immunorecognition as well as neutralization of its toxins by a hetero-specific antivenom. Applying C_18_ reverse-phase high-performance liquid chromatography (RP-HPLC) and tandem mass spectrometry (LC-MS/MS), three-finger toxins (3FTx) were shown to dominate the venom proteome by 90.48% of total venom proteins. Other proteins in the venom comprised snake venom metalloproteinases, phospholipases A_2,_ cysteine-rich secretory proteins, venom nerve growth factors, L-amino acid oxidases and vespryn, which were present at much lower abundances. Among all, short-chain alpha-neurotoxins (SαNTX) were the most highly expressed toxin within 3FTx family, constituting 65.87% of the total venom proteins. The SαNTX is the sole neurotoxic component of the venom and has an intravenous median lethal dose (LD_50_) of 0.18 μg/g in mice. The high abundance and low LD_50_ support the potent lethal activity of *N. samarensis* venom. The hetero-specific antivenom, Philippine Cobra Antivenom (PCAV, raised against *Naja philippinensis*) were immunoreactive toward the venom and its protein fractions, including the principal SαNTX. In efficacy study, PCAV was able to cross-neutralize the lethality of SαNTX albeit the effect was weak with a low potency of 0.20 mg/ml (defined as the amount of toxin completely neutralized per milliliter of the antivenom). With a volume of 5 ml, each vial of PCAV may cross-neutralize approximately 1 mg of the toxin *in vivo*. The findings support the potential para-specific use of PCAV in treating envenomation caused by *N. samarensis* while underscoring the need to improve the potency of its neutralization activity, especially against the highly lethal alpha-neurotoxins.

## Introduction

Each year, about 5.4 million snakebites occur worldwide, resulting in 1.8–2.7 million cases of envenomation. Consequently, 81,000 to 138,000 people die due to the toxic effects of snake venom, with three times as many continue to suffer long-term complications of various physical and mental sequelae (Kasturiratne et al., 2008; World Health Organization [WHO], 2019). South Asia, sub-Saharan Africa and Southeast Asia are the regional trio that features the highest prevalence and mortality of snakebite envenomation. This public health crisis heavily affects the impoverished populations in the rural areas, where health resources are scarce, venomous snakes are abundant and human-snake interaction is common due to extensive agricultural practice (World Health Organization [WHO], 2016; Gutiérrez et al., 2017; Ralph et al., 2019).

In Asia, cobras (*Naja* spp.) are the quintessential venomous snakes, typified by their lethal and incapacitating bite. Cobra bite envenomation causes neuromuscular paralysis that leads to respiratory failure, multiple organ failure and death, if proper treatment is unavailable (Tan and Tan, 2016; Thatoi et al., 2016; World Health Organization [WHO], 2016). Also, extensive tissue necrosis can take place at the bite site, resulting in amputation or severe physical deformity and compromising the victim’s quality of life (Mehta and Sashindran, 2002; Jayawardana et al., 2018). The clinical syndrome observed is largely attributed to the neurotoxins (causing paralysis) and cytotoxins (causing tissue necrosis) found abundantly in cobra venoms (Tan et al., 2017c; Wong et al., 2018). Nevertheless, cobra venoms can vary substantially between different species (inter-species venom variation) and even within the same species (intra-species venom variation) due to factors of geographical distribution, ontogenic development and sex of the snake (Mackessy, 2009; Tan et al., 2015d; Huang et al., 2015; Wong et al., 2016; Dutta et al., 2017). Variation in the composition and antigenicity of snake venom has important medical implications as it can result in variable venom toxicity and inconsistent effectiveness of antivenom therapy. Moreover, species-specific antivenoms are still lacking in many regions.

**FIGURE 1:**
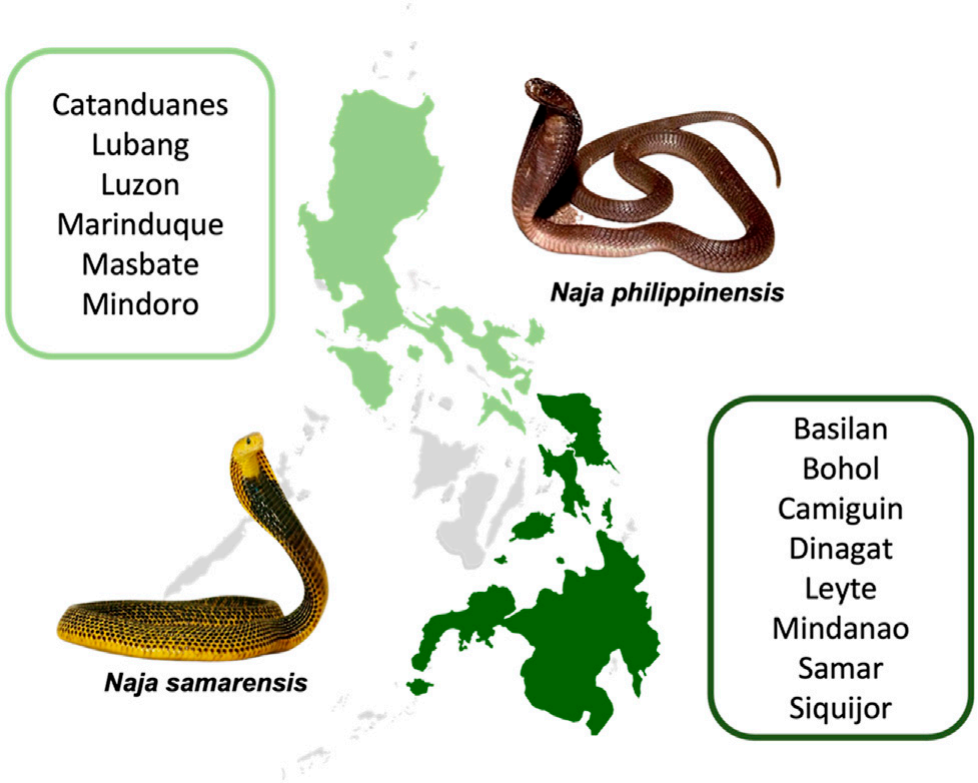
Geographical distribution of the Northern Philippine Cobra (*Naja philippinensis*) and Samar Cobra or Southern Philippine Cobra (*Naja samarensis*). Regions shaded in green are where the cobras are native to: *N. philippinensis* (light green); *N. samarensis* (dark green). Words in green boxes next to the shaded areas refer to islands in the Philippines where the respective cobra species can be found (Sy and Mangkabong, 2018; Sy and Bucol, 2020; Uetz et al., 2021a; Uetz et al., 2021b).

It is therefore crucial to characterize the venom properties of different snake species, addressing the unique protein composition and antigenicity therein. In the Philippines, there are two endemic cobra species in the country: the Philippine cobra (*Naja philippinensis*) which is distributed in the northern Philippines, and the Samar cobra (*Naja samarensis*) that can be found in the southern Philippines (Wüster and Thorpe, 1990; Sanguila et al., 2016; Sy and Mangkabong, 2018). Unlike the Philippine Cobra, the Samar Cobra (*N. samarensis*, also known as Visayan cobra or Peters’ cobra) is lesser known perhaps owing to insufficient epidemiological data and the lack of study on its venom properties. The Samar Cobra is distributed in the southern part of the Philippines, as in the islands of Visayas (Bohol, Leyte and Samar), Mindanao, Camiguin, Dinagat, Basilan and Siquijor (Sanguila et al., 2016) (Figure 1). *N. samarensis* can grow up to 1.4 m in length, and it displays striking body coloration of black and bright yellow, in contrast to *N. philippinensis* which is usually light to olive brown (Avilonzoo, 2015) (Figure 1). Envenomation caused by *N. samarensis* has been anecdotally reported to cause severe neurotoxicity somewhat similar to that caused by *N. philippinensis*, manifested by rapid neuromuscular paralysis that leads to respiratory failure and death (Watt et al., 1988; World Health Organization [WHO], 2018). As a spitting cobra, it is also capable of spitting (or spraying) venom into the eyes of aggressors, resulting in venom ophthalmia (Chu et al., 2010; Chang et al., 2020).

Although antivenom is the only definitive treatment for snakebite envenomation, there is however, no antivenom specific for *N. samarensis* available currently. The Research Institute for Tropical Medicine (RITM) in the Philippines does produce an antivenom raised against *N. philippinensis*, *i.e.*, the Philippine Cobra Antivenom (PCAV) for domestic use, mainly in the northern region of the country (Dimaano, 2018). The supply of PCAV is, however, limited and its clinical use for *N. samarensis* envenomation has not been well established. In a preclinical study, Tan et al. (2021) recently showed that PCAV weakly cross-neutralized the lethal effect of *N. samarensis* venom in mice with a low potency. Presumably, the cross-neutralization efficacy of PCAV was limited by variation in the *N. samarensis* venom composition and toxin antigenicity. Hence, applying a protein decomplexation strategy, the present study investigated the venom proteome of *N. samarensis* to unravel the venom composition and toxins implicated in envenomation. The immunoreactivity and neutralization efficacy of PCAV against the toxins were also examined for insights into the optimization of antivenom production in the region.

## Materials and Methods

### Venom and Antivenoms

The venom sample of *Naja samarensis* was pooled from the milking of multiple adult specimens (*n* = 5) from the southern Philippines. The *N. philippinensis* and *Calloselasma rhodostoma* venoms were supplied by Latoxan Venom Supply (Valence, France) and Queen Saovabha Memorial Institute (QSMI, Thailand), respectively. All venom samples were kept in lyophilized form at −20°C until use. The antivenom used in this work was the Philippine Cobra Antivenom (PCAV; batch no.: 201804; expiry date: April 2021). PCAV is a monovalent antivenom raised against the venom of *N. philippinensis*, a product from Research Institute for Tropical Medicine (RITM), the Philippines. PCAV was used before its expiration date.

### Determination of Protein Concentration of Venoms and Antivenom

Protein concentration of venoms and toxins was determined using Nanodrop™ 2000 spectrophotometer (Waltham, MA, United States). Protein concentration of PCAV was determined with Thermo Scientific™ Pierce™ Bicinchoninic Acid (BCA) Protein Assay kit (Rockford, IL, United States) with bovine serum albumin used as protein standard in calibration.

### Chemicals and Materials

Ammonium bicarbonate, dithiothreitol (DTT) and iodoacetamide (IAA) were purchased from Sigma-Aldrich (St. Louis, MO, United States). MS grade trypsin protease and HPLC grade solvents were purchased from Thermo Scientific™ Pierce™ (Rockford, IL, United States). LiChrospher^®^ WP300 RP-18 HPLC column (5 μm particle size) and Millipore ZipTip^®^ C_18_ Pipette Tips were procured from Merck (Burlington, MA, United States). Other chemicals and solvents used were of analytical grade and purchased from Sigma-Aldrich (St. Louis, Missouri, United States).

### Reverse-Phase High-Performance Liquid Chromatography

The crude sample in the form of lyophilized venom (3 mg) was reconstituted in 200 μl ultrapure water and subjected to C_18_ reverse-phase high-performance liquid chromatography (RP-HPLC) at a flow rate of 1 ml/min. The LiChrospher^®^ WP300 RP-18 HPLC column (250 × 4 mm, 5 μm particle size) was pre-equilibrated with Solvent A (0.1% TFA in water) and eluted with Solvent B (0.1% TFA in ACN) in linear gradient setting (5% B for 10 min, 5–15% B for 20 min, 15–45% B for 120 min and 45–70% for 20 min). The eluted peaks were detected at 215 nm and fractions were manually collected, lyophilized and stored at −20°C until use.

### Sodium Dodecyl Sulfate-Polyacrylamide Gel Electrophoresis

Sodium dodecyl sulfate-polyacrylamide gel electrophoresis (SDS-PAGE) was conducted as per Laemmli (Laemmli, 1970). SmoBio PM2700 ExcelBand™ 3-color Broad Range Protein Marker (5–245 kDa) (Hsinchu, Taiwan) was utilized for molecular weight calibration. 25 μg of crude venoms (*N. samarensis* and *N. philippinensis*) and 10 μg of RP-HPLC fractions (eluted proteins) were redissolved in ultrapure water and separated electrophoretically *via* 15% SDS-PAGE under reducing conditions, at 90 V for 2.5 h. Proteins were visualized using Coomassie Brilliant Blue R250.

### In-Solution Tryptic Digestion

The fractions collected from RP-HPLC were subjected to in-solution tryptic digestion as per the protocol previously outlined (Tan et al., 2017a). The venom samples were reduced with DTT, alkylated with IAA, and digested with MS grade trypsin protease. The tryptic peptides were concentrated and desalted with Millipore ZipTip^®^ C_18_ Pipette Tips in accordance with the manufacturer’s recommendations to augment the performance of mass spectrometry.

### Protein Identification by Tandem Mass Spectrometry (Nano-ESI-LCMS/MS)

The trypsin-digested peptides were redissolved in 7 μl of 0.1% formic acid in water and exposed to nano-electrospray ionization MS/MS by adopting Agilent 1200 HPLC-Chip/MS Interface in conjunction with Agilent 6550 Accurate-Mass Q-TOF LC/MS system. Samples were primed in a large capacity chip Zorbax 300 Å, C18, 160 nl enrichment column, 75 μm × 150 mm analytical column and 5 μm particles (Agilent part no. G4240–62010). Running parameters were configured to 1 μl injection volume per sample, 0.4 μl/min flow rate, and the peptides were separated by running Solution B (0.1% formic acid in acetonitrile) in configured linear gradient setting (5–50% B for 11 min, 50%–70% B for 4 min and 70% B for 3 min) using Agilent 1200 series nano-flow LC pump. Positive ionization mode was selected for ion polarity. Flow rate and temperature of drying gas were set to 11 l/min and 290°C, respectively. Fragmentor voltage was configured to 175 V and the capillary voltage was set to 1800 V. Spectra was obtained in a MS/MS mode with a MS scan range of 200–3,000 m/z and MS/MS scan range of 50–3,200 m/z. Precursor charge selection was set as doubly charge state and above with the exclusion of precursors 1221.9906 m/z (z = 1) and 299.2944 (z = 1) set as reference ions. Data were generated with MH (protonated peptide ion) mass span between 50 and 3,200 Da and sorted with Agilent Spectrum Mill MS Proteomics Workbench software packages. Carbamidomethylation of cysteine residues was set as a fixed modification and oxidation of methionine residues as variable modification. The data derived from mass spectrometry was searched against a non-redundant NCBI database of Serpentes (taxid: 8570) integrated with an in-house transcript database containing related elapids (cobras and king cobra) as previously described (Tan et al., 2015a; Tan et al., 2017a; Chong et al., 2019). Protein identification was verified with the following filters: protein score >15 and peptide score >5. Identified proteins were filtered at <1% false discovery rate (FDR).

### Estimation of Relative Abundance of Proteins

The abundance of each individual protein in a fraction was estimated based on the mean spectral intensity (MSI) of its peptides and the peak area under curve (AUC) of its corresponding chromatographic fraction, as described in the formula below:

$$Relative\;abundance\;of\;protein\;A\;in\;fraction\;B\;=\;\frac{MSI\;of\;protein\;A\;in\;HPLC\;fraction\;B}{Total\;MSI\;of\;all\;proteins\;in\;HPLC\;fraction\;B}\;\times AUC\;of\;the\;HPLC\;fraction\;B\left(\%\right)$$

The MSI of protein A in HPLC-derived fraction B is the mean spectral intensity of the peptide ions assigned to protein A eluted from fraction B. The peak area under curve (AUC) was determined from the chromatogram using the Shimadzu LC Solution Software (Shimadzu, Kyoto, Japan).

### ELISA Immunoprofiling of Venom Fractions

The immunological binding properties between antivenom and venom fractions were tested with an indirect enzyme-linked immunosorbent assay (ELISA) as described by Tan et al. (2015c). First, 96-well immunoplate were pre-coated overnight with 10 ng of whole venoms and RP-HPLC-derived fractions of *N. samarensis* at 4°C. *N. philippinensis* and *C. rhodostoma* venoms were served as positive and negative controls, respectively. Next day, the wells were flicked dried and repeatedly washed four times with phosphate-buffered saline containing 0.5% Tween^®^20 (PBST). Antivenom (PCAV) was prepared at a stock concentration of 10 mg/ml and further diluted as needed. 100 μl of the properly diluted antivenom (1:450) in PBST was added into venom-coated wells, followed by incubation for 1 h at room temperature. The wells were then added with horseradish peroxidase-conjugated anti-horse-IgG in PBST (1:10000) and incubated for another hour at room temperature. The wells were washed four times with PBST before adding 50 μl freshly prepared 3,3′,5,5′-Tetramethylbenzidine (TMB) substrate. The plate was left for 25 min at room temperature in the dark for enzymatic reaction to take place. The reaction was then terminated by adding 50 μl of 2 M sulfuric acid per well, and the absorbance was read at 450 nm against a blank. Values presented were the means ± S.E.M. of triplicates.

### Determination of Lethality of Venom Toxins and the Neutralization by Antivenom

The median lethal doses (LD_50_) of the principal toxins in *N. samarensis* venom (Fraction 1 from the RP-HPLC, which was found lethal following the lethality screening of various fractions) and in *N. philippinensis* venom (Fraction 1, as identified according to Tan et al. (2019)) were determined in ICR albino mice (*n* = 4 per dose, 20–25 g), supplied by the Animal Experimental Unit, University of Malaya. The experiment procedure was approved by the Institutional Animal Care and Use Committee of the university (reference: 2019-220108/PHAR/R/TCH).

In brief, various doses of the toxins were prepared in 100 µl and injected intravenously into the mice via caudal vein. The mice were closely monitored and allowed free access to food and water *ad libitum*. The survival ratio was recorded 24 h post-injection for determination of LD_50_, which is defined as the toxin dose (µg of venom/g of mice) at which 50% of the mice were dead. In antivenom neutralization study, different doses of PCAV were mixed with the respective toxins at a challenge dose of 5 LD_50_ each, dissolved in a total volume of 250 µl and incubated at 37°C for 30 min. The pre-incubated mixture was then injected intravenously into the mice (*n* = 4 per dose, 20–25 g). The neutralization was determined by median effective dose (ED_50_), defined as the volume dose of antivenom (µl) at which 50% of the mice survived. Both LD_50_, ED_50_ (Morais et al., 2010) and the 95% confidence intervals were calculated by the Probit analysis (Finney and Tattersfield, 1952), using BioStat 2009 analysis software (AnalystSoft Inc., Canada).

The neutralizing capacity of PCAV was further expressed as median effective ratio (ER_50_), defined as the ratio of venom or toxin (mg) to the volume of antivenom (ml) at which 50% of mice survived) and potency (P), defined as the amount of venom or toxin (mg) completely neutralized per unit volume (ml) of antivenom (thus 100% survival). The potency (P) was normalized by the PCAV concentration to obtain normalized potency (n-P), defined as the amount of venom or toxin (mg) completely neutralized per unit amount of antivenom protein (g) (Tan and Tan, 2019).

### Data availability

The mass spectrometry proteomics data have been deposited to the ProteomeXchange Consortium *via* the iProX partner repository (https://www.iprox.org) (Ma et al., 2019) with the dataset identifier PXD026270, subproject ID: IPX0003097001.

## Results and Discussion

### Chromatographic and Electrophoretic Profiles of *Naja samarensis* Venom

**FIGURE 2:**
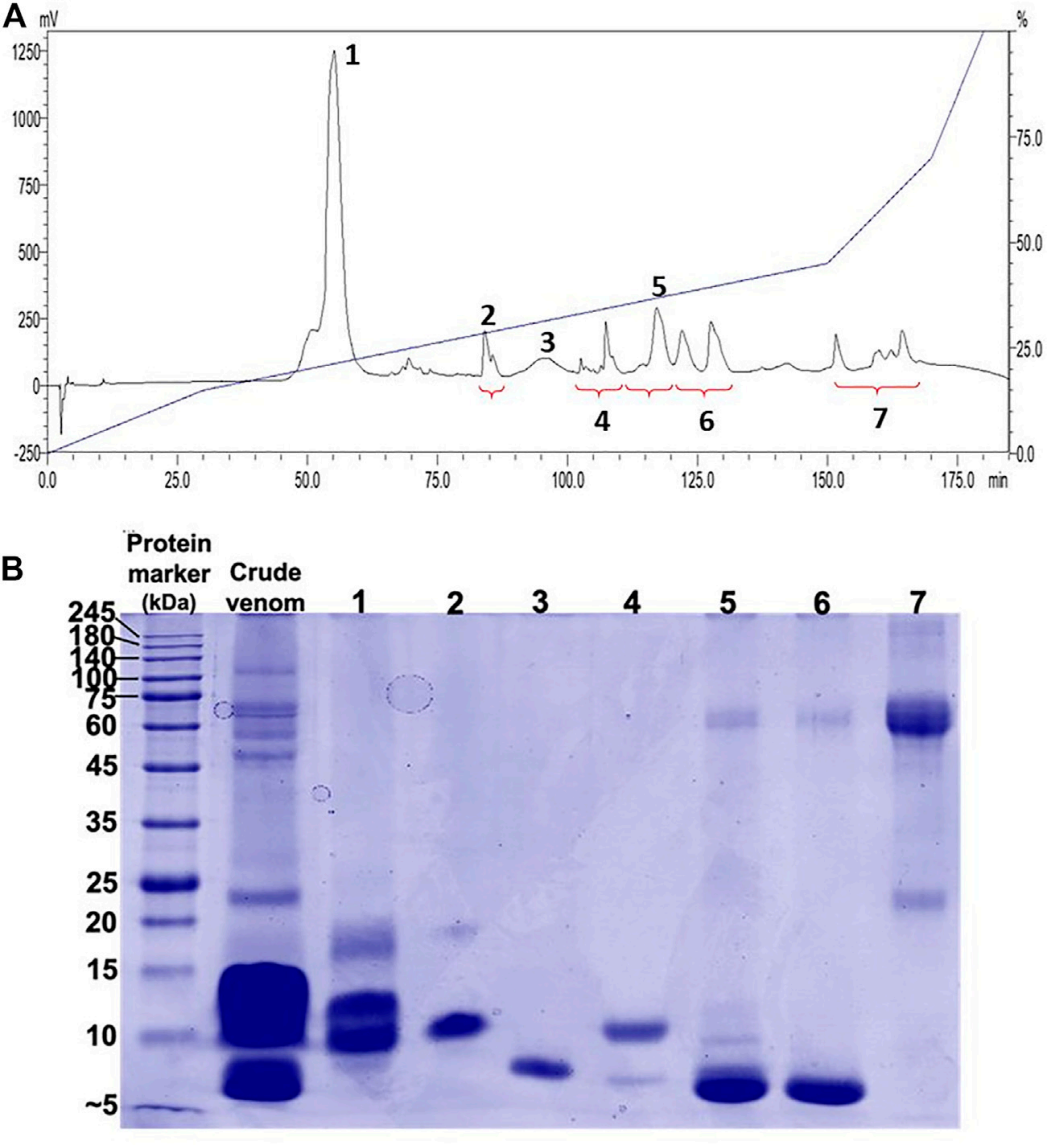
Chromatographic and electrophoretic profiles of *Naja samarensis* venom. **(A)** Upper panel: C_18_ reverse-phase high-performance liquid chromatography (RP-HPLC) of venom. **(B)** Lower panel: 15% polyacrylamide gel electrophoresis (SDS-PAGE) profile of RP-HPLC eluted fractions electrophoresed under reducing conditions.

Reverse-phase HPLC (RP-HPLC) resolved *N. samarensis* venom into multiple peaks that were grouped into seven fractions (Figure 2). As depicted by SDS-PAGE in Figure 2, the majority of venom proteins eluted from fractions 1 to 6 amid chromatography (corresponding to the elution time between 50 and 130 min) were low molecular weight proteins (<20 kDa), consistent with low hydrophobicity of protein. The medium (>25–35 kDa) and high (>35 kDa) molecular weight proteins were present more predominantly in fraction seven which was eluted between 150 and 170 min. Low molecular weight proteins constituted more than 95% of the total venom proteins based on the calculation of chromatographic peak area.

**FIGURE 3:**
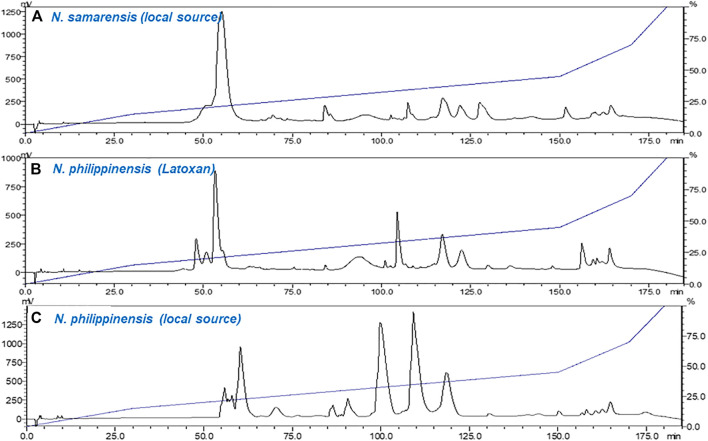
Chromatograms of crude venom samples fractionated using C_18_ reverse-phase high-performance liquid chromatography (RP-HPLC). **(A)** *Naja samarensis* (local source); **(B)** *Naja philippinensis* (captive source from Latoxan); and **(C)** *Naja philippinensis* (local source). Red dotted lines highlight the elution between 80 and 140 min.

The venom chromatogram (C_18_ RP-HPLC) of *N. samarensis* in this study was further compared with that of *N. philippinensis* from a captive source (Latoxan) and a wild-caught specimen. Under the same experimental conditions, the venoms of *N. samarensis* and *N. philippinensis* showed similar patterns of protein separation (Figure 3). RP-HPLC profiling of the venoms showed the elution of predominantly low molecular weight neurotoxins between 45 and 80 min (Figure 3). Between 80 and 140 min, the elution profiles of *N. samarensis* venom and the commercially supplied *N. philippinensis* venom (Latoxan) were comparable, whereas variation was noticed in the wild-caught *N. philippinensis* venom in which proteins eluted had a higher abundance (based on area under curve). The intra-specific venom variability in *N. philippinensis* observed is probably related to habitat and availability of specific preys (dietary conditions), as previously it has been established that venom compositions can be greatly influenced by the diets of snake (Daltry et al., 1996; Pahari et al., 2007; Barlow et al., 2009; Harris et al., 2020). Although an earlier study suggested the expression of venom proteins is unlikely to be affected by their external housing enclosures (captive and wild-caught) (McCleary et al., 2016), minor differences in their expression abundances was notable. In *N. samarensis* venom, the dominance of low molecular weight proteins is similar to the venom of *N. philippinensis* (Tan et al., 2019b), and several other medically important Asiatic cobras reported including *N. kaouthia* (Tan et al., 2015d; Xu et al., 2017)*, Naja siamensis* (Liu et al., 2017), *Naja sputatrix* (Tan et al., 2017c)*, Naja atra* (Huang et al., 2015) and *Naja naja* (Sintiprungrat et al., 2016; Chanda et al., 2018; Wong et al., 2018). Consistently, high molecular weight proteins constituted minor components in these cobra venoms, with a relative protein abundance of less than 10%.

### Venom Proteome of *Naja samarensis*

**FIGURE 4:**
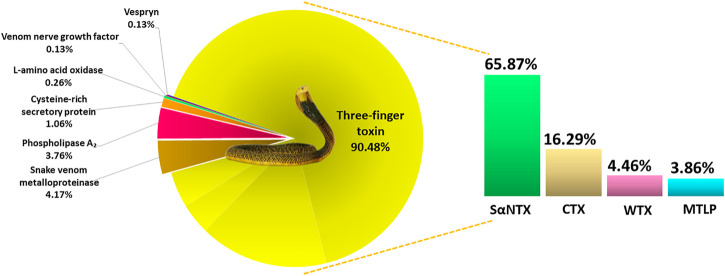
Venom proteome of *Naja samarensis* classified according to the toxin protein families, with relative abundances (%) based on HPLC profile and relative spectral intensity from mass spectrometry analysis (as described in the method section). Abbreviations: CTX, cytotoxin; MTLP, muscarinic toxin-like protein; SαNTX, short-chain alpha-neurotoxin; WTX, weak toxin.

Using tandem mass spectrometry, proteins in the RP-HPLC fractions of *N. samarensis* venom were identified and shown as in Table 1. In total, 31 non-redundant proteins were identified from the seven RP-HPLC fractions. These proteins were further classified into seven protein families of typical snake venom toxins at varying relative protein abundances (Figure 4). Three-finger toxins (3FTx) were the most abundantly and diversely expressed proteins, comprising 16 distinct proteins that constitute 90.48% of total venom proteins, and followed by snake venom metalloproteinases (SVMP, 4.17%) and phospholipases A_2_ (PLA_2_, 3.76%). The remaining minor toxins collectively accounted for <2% of total venom proteins; these were toxin families of cysteine-rich secretory protein (CRiSP, 1.06%), L-amino acid oxidase (LAAO, 0.26%), venom nerve growth factor (vNGF, 0.13%) and vespryn (VES, 0.13%) (Figure 4). The proteins identified were consistent with the chromatographic and electrophoretic profiles of the venom, where low MW proteins including three-finger toxins (3FTx) and phospholipases A_2_ (PLA_2_) were eluted mainly from fraction 1 to fraction 6 of the RP-HPLC, followed by medium and high MW proteins from fraction 7 (Figure 2). The data of mass spectrometry analysis including the spectral mass/charge values, protein scores and peptide sequences were provided in Supplemental File.

**TABLE 1:** Protein identification from *Naja samarensis* venom fractions isolated by reverse-phase high-performance liquid chromatography using nano-ESI-LCMS/MS.

RP-HPLC derived fractions/Protein name	Database accession	Species annotated	Protein score	Relative abundance (%)
Fraction 1				**65.87**
Short neurotoxin 1	P60774	*N. samarensis*	96.24	27.93
Short neurotoxin 1	P01424	*N. melanoleuca*	42.31	34.87
Cobrotoxin	P60770	*N. atra*	22.22	3.07
Fraction 2				**3.86**
Muscarinic toxin-like protein 2	P82463	*N. kaouthia*	46.73	3.86
Fraction 3				**4.46**
Weak toxin CM-2	P01415	*N. haje*	17.79	4.46
Fraction 4				**3.98**
Acidic phospholipase A2 natratoxin	A4FS04	*N. atra*	68.39	1.93
Acidic phospholipase A2 D	Q9I900	*N. sputatrix*	57.64	1.83
Cytotoxin 1	P01467	*N. mossambica*	40.65	0.11
Cytotoxin 3	P01470	*N. mossambica*	37.27	0.11
Fraction 5				**6.93**
Cytotoxin 2	P01441	*N. oxiana*	162.93	1.86
Cytotoxin 10	P86541	*N. naja*	78.78	0.65
Cytotoxin 2	P01445	*N. kaouthia*	74.29	0.37
Cytotoxin SP15c	P60308	*N. atra*	73.38	3.26
Cytotoxin homolog	P14541	*N. kaouthia*	59.57	0.15
Cytotoxin 1	P01467	*N. mossambica*	50.54	0.27
Venom nerve growth factor 2	Q5YF89	*N. sputatrix*	30.51	0.13
Hemextin A	P0DQH3	*H. haemachatus*	18.59	0.23
Cysteine-rich venom protein ophanin	Q7ZT98	*O. hannah*	16.61	0.01
Fraction 6				**9.36**
Cytotoxin 2	P01441	*N. oxiana*	108.69	1.00
Cytotoxin 2a	P86538	*N. naja*	62.12	1.74
Cytotoxin 1	P01447	*N. naja*	52.31	5.87
Cytotoxin 1	P01467	*N. mossambica*	30.39	0.70
Zinc metalloproteinase-disintegrin-like atrase-B	D6PXE8	*N. atra*	24.83	0.07
Fraction 7				**5.54**
L-amino-acid oxidase	P81383	*O. hannah*	111.89	0.26
Natrin	*CL2736.Contig1_NsM2	*N. atra*	100.47	0.42
Cysteine-rich venom protein natrin-1	Q7T1K6	*N. atra*	78.40	0.33
Cysteine-rich venom protein ophanin	Q7ZT98	*O. hannah*	65.76	0.16
Cysteine-rich venom protein ophanin-like	XP_026568636.1	*P. textilis*	48.83	0.14
Zinc metalloproteinase-disintegrin-like atrase-A	D5LMJ3	*N. atra*	79.58	0.21
Metalloproteinase atrase A	*CL115.Contig7_NkT	*N. atra*	41.01	0.20
Metalloproteinase	B5G6F7	*D. vestigiata*	49.02	0.15
Zinc metalloproteinase-disintegrin-like MTP4	F8RKW1	*D. coronoides*	19.50	1.22
Zinc metalloproteinase-disintegrin-like atrase-B	D6PXE8	*N. atra*	22.21	2.22
Hemorrhagic metalloproteinase-disintegrin-like kaouthiagin	P82942	*N. kaouthia*	22.92	0.10
Ohanin	P83234	*O. hannah*	20.66	0.13

Relative abundance (%) refers to the abundance of individual proteins in a fraction, which was estimated based on the mean spectral intensity (MSI) of the peptide ions and the peak area under curve of its corresponding chromatographic fractions. Bold values are subtotals of protein abundances per fraction.

*Protein codes with suffix “_NsM2” and “_NkT” indicate venom proteins identified based on tryptic peptides matched to sequence from an in-house transcript database containing RNAseq, specific for the following: NsM: Naja sumatrana, Malaysia and NkT: Naja kaouthia, Thailand. Mass spectrometric data and peptide sequences are available in Supplemental file.

Abbreviations: D. coronoides, Drysdalia coronoides; D. vestigiata, Demansia vestigiata; H. haemachatus, Hemachatus haemachatus; N. atra, Naja atra; N. haje, Naja haje; N. kaouthia, Naja kaouthia; N. melanoleuca, Naja melanoleuca; N. mossambica, Naja mossambica; N. naja, Naja naja; N. oxiana, Naja oxiana; N. samarensis, Naja samarensis; N. sputatrix, Naja sputatrix; NCBI, National Centre for Biotechnology Information; O. hannah, Ophiophagus hannah; P. textilis, Pseudonaja textilis; RP-HPLC, reverse-phase high performance liquid chromatography.

Based on the relative abundances of the proteins, 3FTxs in *N. samarensis* venom are composed of diverse isoforms of short-chain alpha-neurotoxin (SαNTX), cytotoxin (CTX), muscarinic toxin-like protein (MTLP) and weak toxin (WTX), accounting for 90.48% of the total venom proteins. Among these, SαNTX is the sole and most abundantly expressed subclass of alpha-neurotoxin (αNTX) identified in the venom, accounting for 65.87% of the total venom proteins. In total, three SαNTX forms were present (Table 2). Cobra venoms, in general, exhibit a common phenotype characterized by dominant expression of either short-chain or long-chain alpha-neurotoxins (SαNTX or LαNTX). Interestingly, the *N. samarensis* venom proteome has only SαNTX as its alpha-neurotoxins whereas LαNTX was absent—a feature similar to that observed in the venom proteome of *N. philippinensis* (Tan et al., 2019b), an allopatric cobra species distributed in the northern islands. The lack of LαNTX has also been noted in the venom proteomes of the monocled cobra (*N. kaouthia*) from Vietnam (Tan et al., 2015d) and the Chinese cobra (*Naja atra*) from mainland China (Shan et al., 2016). These cobras, representing far eastern dispersal of *Naja* species, probably evolved a specialized toxin arsenal that fully deploys SαNTX in predation. Nevertheless, the expression levels of SαNTX vary between these species, with *N. samarensis* recording the highest SαNTX abundance (65.87% of total venom proteins, current work), followed by *N. philippinensis* (44.55%) (Tan et al., 2019b), *N. atra* (southern China Mainland) (11.2%) (Shan et al., 2016) and *N. kaouthia* (Vietnam) (9.2%) (Tan et al., 2015d). The apparent variance in SαNTX content is reflected in the differential lethal potency of the venoms, as discussed below.

**TABLE 2:** Relative abundance of different toxin families identified from *Naja samarensis* venom.

Protein family/Name	Fraction(s)	Database accession	Species	Relative abundance (%)
Three-finger toxin (3FTx)				**90.48**
Short-chain alpha-neurotoxin (SαNTX)				**65.87**
Short neurotoxin 1	1	P01424	*N. melanoleuca*	34.87
Cobrotoxin	1	P60770	*N. atra*	3.07
Short neurotoxin 1	1	P60774	*N. samarensis*	27.93
Cytotoxin (CTX)				**16.29**
Cytotoxin 1	6	P01467	*N. mossambica*	0.70
Cytotoxin 2	5,6	P01441	*N. oxiana*	2.84
Cytotoxin 2	5	P01445	*N. kaouthia*	0.37
Cytotoxin 1	6	P01447	*N. naja*	5.87
Cytotoxin 1	4,5	P01467	*N. mossambica*	0.38
Cytotoxin 3	4	P01470	*N. mossambica*	0.11
Hemextin A	5	P0DQH3	*H. haemachatus*	0.23
Cytotoxin homolog	5	P14541	*N. kaouthia*	0.15
Cytotoxin SP15c	5	P60308	*N. atra*	3.26
Cytotoxin 2a	6	P86538	*N. naja*	1.74
Cytotoxin 10	5	P86541	*N. naja*	0.65
Muscarinic toxin-like protein (MTLP)				**3.86**
Muscarinic toxin-like protein 2	2	P82463	*N. kaouthia*	3.86
Weak toxin (WTX)				**4.46**
Weak toxin CM-2	3	P01415	*N. haje*	4.46
Snake venom metalloproteinase (SVMP)				**4.17**
Metalloproteinase	7	B5G6F7	*D. vestigiata*	0.15
Zinc metalloproteinase-disintegrin-like atrase-A	7	D5LMJ3	*N. atra*	0.21
Zinc metalloproteinase-disintegrin-like atrase-B	6,7	D6PXE8	*N. atra*	2.29
Zinc metalloproteinase-disintegrin-like MTP4	7	F8RKW1	*D. coronoides*	1.22
Hemorrhagic metalloproteinase-disintegrin-like kaouthiagin	7	P82942	*N. kaouthia*	0.10
Metalloproteinase atrase A	7	*CL115.Contig7_NkT	*N. atra*	0.20
Phospholipase A_2_ (PLA_2_)				**3.76**
Acidic phospholipase A2 natratoxin	4	A4FS04	*N. atra*	1.93
Acidic phospholipase A2 D	4	Q9I900	*N. sputatrix*	1.83
Cysteine-rich secretory protein (CRiSP)				**1.06**
Cysteine-rich venom protein natrin-1	7	Q7T1K6	*N. atra*	0.33
Cysteine-rich venom protein ophanin	5,7	Q7ZT98	*O. hannah*	0.17
Cysteine-rich venom protein ophanin-like	7	XP_026568636.1	*P. textilis*	0.14
Natrin	7	*CL2736.Contig1_NsM2	*N. atra*	0.42
L-amino-acid oxidase (LAAO)				**0.26**
L-amino-acid oxidase	7	P81383	*O. hannah*	0.26
Venom nerve growth factor (vNGF)				**0.13**
Venom nerve growth factor 2	5	Q5YF89	*N. sputatrix*	0.13
Vespryn (VES)				**0.13**
Ohanin	7	P83234	*O. hannah*	0.13

The percentages (%) refers to the relative abundances of proteins identified from the HPLC-derived venom fractions. Bold values are subtotals of protein abundances per toxin family or subfamily.

*Protein accession numbers with suffixes “_NsM2” and “_NkT” indicate proteins matched to sequences from an in-house transcript database containing RNAseq, of the following cobras: NsM: Naja sumatrana, Malaysia and NkT: Naja kaouthia, Thailand. Mass spectrometric data and peptide sequences are available in Supplemental File.

Abbreviations: D. vestigiata, Demansia vestigiata; D. coronoides, Drysdalia coronoides; H. haemachatus, Hemachatus haemachatus; N. atra, Naja atra; N. haje, Naja haje; N. kaouthia, Naja kaouthia; N. melanoleuca, Naja melanoleuca; N. mossambica, Naja mossambica; N. naja, Naja naja; N. oxiana, Naja oxiana; N. samarensis, Naja samarensis; N. sputatrix, Naja sputatrix; O. hannah, Ophiophagus hannah; P. textilis, Pseudonaja textilis.

In cobra bite envenomation, the alpha-neurotoxins block post-synaptic nicotinic acetylcholine receptors (nAChR) at the neuromuscular junctions, thereby inhibiting neurotransmission and causing paralysis (Barber et al., 2013). These toxins are typically the principal lethal components in the venoms of most cobra and sea snake species (Tan et al., 2016b; Wong et al., 2016; Tan et al., 2017c; Silva et al., 2018; Tan et al., 2019a; Harris et al., 2020). Previous studies indicated that short neurotoxins (SαNTX), in contrast to LαNTX, bind less irreversibly to nAChR and are therefore, theoretically, “less lethal” with low medical importance (Chicheportiche et al., 1975; Utkin, 2013). In lethality test, however, both SαNTX and LαNTX induced comparable neurotoxicity *in vivo*, and are highly lethal to mice with median lethal doses (LD_50_) ranging between 0.05 and 0.25 μg/g (Tan et al., 2015b; Tan et al., 2016b; Wong et al., 2016; Tan et al., 2017a; Tan et al., 2019a). The lethal potency of cobra venom has been shown to correlate with the abundance of alpha-neurotoxins (both SαNTX and LαNTX) in the venom (Tan et al., 2019b). Comparing the aforementioned eastern cobras whose venom neurotoxicity is exclusively SαNTX-driven, we observed a trend that supports the correlation between the differential SαNTX expression, and the lethality induced. The highly lethal *N. samarensis* and *N. philippinensis* venoms (with intravenous LD_50_ between 0.1 μg/g and 0.2 μg/g) were dominated by SαNTX up to 50–70% (Tan et al., 2019b; Tan et al., 2021); while in the Chinese *N. atra* and Vietnamese *N. kaouthia* venoms (intravenous LD_50_ is ∼0.9 μg/g), the lethality is approximately 5-fold weaker, consistent with their much lower expression (by ∼6-fold) of SαNTX (∼9%) in the respective venoms (Tan et al., 2015d; Ratanabanangkoon et al., 2016). The finding in the present work indicates that the neurotoxicity of *N. samarensis* venom toxicity is principally driven by the abundant SαNTX and underscores the need for antivenom treatment that can neutralize the toxin effectively.

Cytotoxins (CTX, including cytotoxin-like homologs) made up 16.29% of the total venom proteins in *N. samarensis* (Figure 4). CTX is the second most abundantly expressed 3FTx, with 11 distinct subtypes identified in the proteome (Table 2). Contrary to alpha-neurotoxins, CTX is generally less lethal with intravenous LD_50_ values higher than 1.0 μg/g in mice (Leong et al., 2015; Tan et al., 2016b; Wong et al., 2016; Tan et al., 2017c). CTX with membrane-damaging disposition is implicated in the local envenoming effect of cobras for tissue necrosis and venom ophthalmia (Chu et al., 2010; Tan and Tan, 2016; Chang et al., 2020). CTX usually constitutes the major subtype of 3FTx in most cobra venoms, accounting for at least 20–72% of total venom proteins (Huang et al., 2015; Laustsen et al., 2015; Tan et al., 2015d; Shan et al., 2016; Sintiprungrat et al., 2016; Wong et al., 2016; Tan et al., 2017c). The lower abundance of CTX in *N. samarensis* venom (current work) is exceptional, as with the proteomic abundance of CTX in *N. philippinensis* venom (q.v. Table 3 for comparison). Together, the observation suggests that the two endemic cobra species in the Philippines exhibit a unique venom phenotype characterized by reduced expression of CTX and high abundance of SαNTX. The finding is also consistent with clinical envenomation caused by the Philippine cobras, in which local tissue necrotic effect is rare while neurotoxicity is prominent (Watt et al., 1988). On the other hand, other eastern Asiatic cobra species, exemplified by *N. atra* (China and Taiwan) and *N. kaouthia* (Vietnam), have venoms that contain high abundances of CTX but fewer alpha-neurotoxins (Tan et al., 2015d; Huang et al., 2015; Shan et al., 2016). Clinically, they produce envenomation effect marked by severe tissue necrosis but little or negligible neurotoxicity—a clinical phenotype opposing to that in envenomation caused by the Philippine cobras. The mechanisms that underlie the evolutionary divergence in their 3FTx compositions and the resultant dichotomous toxicity of the venoms remain unknown, but presumably are related to the paleo-geographical separation of the Philippine islands from Taiwan in the north (by Luzon Strait) and the Sunda Shelf (rest of Southeast Asia) in the west (by South China Sea). Hence, the overall management for envenomation caused by cobras in the region involving antivenom use and/or supportive treatment, cannot be generalized and should be strategized according to the venom’s principal toxicity of different species.

**TABLE 3:** Comparison of venom toxin families of *Naja samarensis* and *Naja philippinensis*.

Toxin families	*Naja samarensis*	*Naja philippinensis* *^a^*
Localities	Southern Philippines	Northern Philippines
Venom source	Pooled sample (5 adults)^b^	Pooled sample (6 adults)^c^
Fractionation method	Reverse-phase HPLC	Reverse-phase HPLC
Toxin identification method	Nano-ESI-LCMS/MS	Nano-ESI-LCMS/MS
Toxin family abundance (%)		
3FTx	90.48	66.64
SαNTX	65.87	44.55
LαNTX	ND	ND
CTX	16.29	21.31
MTLP	3.86	0.77
WTX	4.46	0.01
PLA_2_	3.76	22.88
SVMP	4.17	3.93
CRiSP	1.06	1.49
LAAO	0.26	0.42
NGF	0.13	0.06
VES	0.13	0.05
CVF	ND	2.38
5′NUC	ND	0.55
PDE	ND	0.45
SVSP	ND	0.35

a Referenced from Tan et al. (2019b).

b Wild caught.

c Supplied by Latoxan Venom Supply, Valence, France.

Abbreviations: 3FTx, Three-finger toxin; 5′NUC, 5′ nucleotidase; CRiSP, cysteine-rich secretory protein; CTX, cytotoxin; CVF, cobra venom factor; HPLC, high-performance liquid chromatography; LAAO, L-amino acid oxidase; LαNTX, long-chain alpha-neurotoxin; MTLP, muscarinic toxin-like protein; ND, not detected; NGF, nerve growth factor; PDE, phosphodiesterase; PLA_2_, phospholipase A_2_; SαNTX, short-chain alpha-neurotoxin; SVMP, snake venom metalloproteinases; SVSP, snake venom serine protease; VES, vespryn; WTX, weak toxin.

Cumulatively, other toxin families made up less than 10% of the total proteins in *N. samarensis* venom (Figure 3). Of these, snake venom metalloproteinases (SVMP) constituted 4.17% of the total venom proteins, comprising six distinct protein forms (Table 2). The SVMP proteins identified belong to the P-III subclass with peptides containing the disintegrin and cysteine-rich domains (Fox and Serrano, 2008). In agreement with existing venom proteomic data of snakes (where details on protein subtypes and abundances were reported), the SVMP in cobra (*Naja* spp.) venoms are typically P-III subclass, and present in trace amount (Tan et al., 2015d; Sintiprungrat et al., 2016; Tan et al., 2017c; Dutta et al., 2017; Chanda et al., 2018; Wong et al., 2018; Tan et al., 2019b). Similarly, SVMP abundance in venom proteome is typically low in Asian elapids including the kraits (*Bungarus* spp.) (Oh et al., 2017; Oh et al., 2019; Hia et al., 2020) and sea snakes (Tan et al., 2015b; Tan et al., 2019a), with the exceptions of king cobra (Tan et al., 2015a; Liu et al., 2017) and Asiatic coral snakes (Tan et al., 2016a; Tan et al., 2019d). The pathophysiological role of SVMP in *Naja* cobra venoms may be related to inflammation and cytotoxicity, while the exact function remains to be elucidated.

The PLA_2_ identified in *N. samarensis* venom proteome (3.76% of total venom proteins) are homologous to the acidic PLA_2_ of *N. kaouthia* and *N. naja*. The acidic PLA_2_ annotated are non-lethal but may contribute to local envenomation effect (Tan et al., 2016b; Wong et al., 2016). In cobra venoms, we previously demonstrated that PLA_2_ follows a unique trend of distribution across the different cobra subgenera, in which the species of *Uraeus* subgenus (African non-spitting cobras) have none to negligible amount of snake venom PLA_2_, while the PLA_2_ abundance is variably higher in the other subgenera (*Naja*, *Afronaja* and *Boulengerina*), especially the spitting cobras presumably associated with algesic (pain-inducing) properties for defense (Tan et al., 2019c). A recent study showed that cobras of spitting lineages have upregulated PLA_2_ to potentiate CTX in activating mammalian sensory nerves, inferring that PLA_2_ expression is indicative of convergent evolution of pain-inducing defensive venoms in the spitting cobras (Kazandjian et al., 2021). The current and previous studies, as shown in Table 3, which analyzed the venom proteomes of the two Asiatic spitting cobras (*N. philippinensis* and *N. samarensis*), reveal an otherwise indication: the two Philippine spitting cobras greatly downregulated the production of PLA_2_ and, to a lesser extent the CTX, while conserving the venom-spitting or venom-spraying trait. The cobra species of far-eastern dispersal in the Philippines thus probably deploy various other toxin components, *e.g.*, SVMP, as above, and L-amino acid oxidases (LAAO, 0.26%), for cytotoxic and pain-inducing properties in venom ophthalmia.

Other minor proteins in *N. samarensis* venom are cysteine-rich secretory protein (CRiSP) (1.06% of total venom protein), venom nerve growth factor (vNGF, 0.13%) and vespryn (0.13%). CRiSP can inhibit the growth of new blood vessels (angiogenesis), increase vascular permeability and thus promote inflammatory responses (leukocyte and neutrophil infiltration) (Yamazaki and Morita, 2004; Mackessy and Heyborne, 2009). Vespryn has been shown to facilitate immobilization of prey by inducing hypolocomotion (Pung et al., 2005; Pung et al., 2006), and vNGF can potentiate venom spread through the release of histamine in the vasculature around the bite site (Kostiza et al., 1995). These are, in general, proteins with higher molecular weights (contrary to the major toxin group, 3FTx) but trace amounts in the venom, implying that they serve ancillary functions without direct lethal implication.

### Immunological Cross-Reactivity of Antivenom and Cross-Neutralization of Venom Toxicity

**FIGURE 5:**
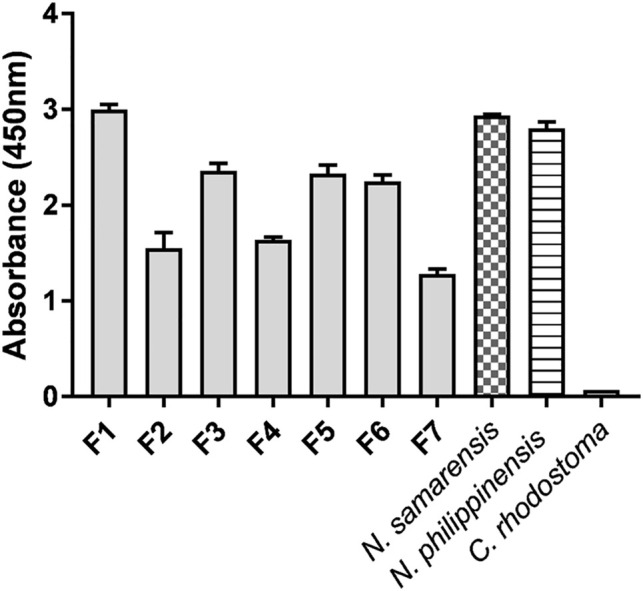
Immunoreactivity of Philippine Cobra Antivenom (PCAV) toward the reverse-phase high-performance liquid chromatography (RP-HPLC) protein fractions (F1-F7) of *Naja samarensis* venom. Values were means ± S.E.M. of triplicates. *Naja philippinensis* and *Calloselasma rhodostoma* venoms were used as positive and negative controls, respectively.

The cross-reactivity of PCAV toward *N. samarensis* venom and its protein fractions separated by RP-HPLC was examined on ELISA (Figure 5). PCAV exhibited comparable immunoreactivity toward the *N. samarensis* venom and the homologous *N. philippinensis* venom, implying the sharing of common toxin epitopes between the two phylogenetically related cobra species. The immunological binding activities of PCAV toward the different protein fractions of *N. samarensis* venom (tested at the same protein amount) were, in general, moderate to strong. High immunoreactivity was observed in fractions 1, 3, 5 and 6 (absorbance values: 2.0–3.0), while the immunoreactivity was moderate in fractions 2, 4 and 7 (absorbance values: 1.0–2.0). Essentially, fraction 1, which formed the major bulk of the venom proteins, showed the highest immunoreactivity with PCAV. Fraction 1 contained the principal toxins, *i.e.*, SαNTX, which are highly neurotoxic and lethal with a low LD_50_ of 0.18 μg/g in mice (Table 4).

**TABLE 4:** Neutralization of the alpha-neurotoxins and venoms of Philippine cobras, *Naja samarensis* and *Naja philippinensis* by antivenom.

		LD_50_ (μg/g)	Challenge dose	PCAV (16.2 ± 1.1 mg/ml)			
				ED_50_ (μl)	ER_50_ (mg/ml)	P (mg/ml)	Normalized P (mg/g)
Alpha-neurotoxins	NS-SαNTX	0.18 (0.12–0.29)	5 LD_50_	91.24 (58.29–142.80)	0.25 (0.16–0.40)	0.20	12.18
	NP-SαNTX	0.10 (0.06–0.15)	5 LD_50_	44.94 (29.60–68.23)	0.28 (0.17–0.42)	0.22	13.74
Venom	Naja samarensis*	0.20 (0.16–0.25)	5 LD_50_	120.86 (104.79–139.40)	0.21 (0.17–0.26)	0.17	10.21
	*Naja philippinensis* ^*#*^	0.18 (0.12–0.27)	5 LD_50_	44.94 (29.60–68.23)	0.50 (0.33–0.75)	0.40	24.72

LD_50_ (Lethal dose): Venom dose (µg/g) at which 50% of the mice were dead.

ED_50_ (Median effective dose): Antivenom dose (μl) at which 50% of tested mice survived.

ER_50_ (Median effective ratio): Antivenom dose (mg/ml) corresponding to ED_50_.

P: Potency of antivenom (mg venom neutralized per ml antivenom).

Normalized P: Potency of antivenom (mg venom neutralized per g antivenom).

Abbreviations: NP-SαNTX, Naja philippinensis short-chain alpha-neurotoxin; NS-SαNTX, Naja samarensis short-chain alpha-neurotoxin; PCAV, Philippine Cobra Antivenom.

^#^ Tan et al., 2019a; (Tan C. H. et al., 2019b).

*Tan et al., 2021; (Tan et al., 2021).

The high immunoreactivity of PCAV toward the principal toxin fraction supports the previous observation of PCAV cross-neutralizing the whole venom of *N. samarensis*, though did not explain its differential neutralization efficacy against *N. philippinensis* and *N. samarensis* venoms, in which the neutralization of the former (homologous venom) was more efficacious (Tan et al., 2021). To elucidate the observation, we isolated the SαNTX from the venoms of *N. samarensis* and *N. philippinensis* based on the RP-HPLC profiles and tested the *in vivo* neutralization activity of PCAV against the toxins from the respective species. The neutralization effects of PCAV against the toxins of *N. samarensis* and *N. philippinensis* were compared in terms of potency, defined as the amount of the toxin completely neutralized per volume dose of the antivenom (mg/ml) (Table 4). It was found that PCAV neutralized the SαNTX of *N. samarensis* and *N. philippinensis* with equipotency (*p* = 0.20 and 0.22 mg/ml, respectively), implying that their SαNTX indeed shared conserved antigenicity that allows immunorecognition and neutralization by the same antivenom. The neutralization potency against the whole venom, however, was lower in *N. samarensis* than *N. philippinensis* by approximately 2-fold. Based on the venom proteomic findings, this could be explained by the higher abundance of SαNTx in the *N. samarensis* venom (65.87%) than in the *N. philippinensis* venom (44.55%) (Table 3), thus requiring a higher dose of antivenom for neutralizing the whole venom toxicity. On the whole, the neutralization potency of PCAV against the SαNTX is low; this is the limiting factor of PCAV efficacy in neutralizing the whole venom toxicity, as shown by the low potency of 0.20–0.40 mg/ml (Table 4). Considering that an adult cobra can readily deliver 50–100 mg dry weight of venom in a bite (author’s venom-milking experience), in the case of complete absorption of 50 mg venom into the systemic circulation, a theoretical dose of 125–250 ml of PCAV which is equivalent to tens of vials of antivenom would be required for complete neutralization in clinical treatment. High doses of antivenom increase the risk of hypersensitivity reactions (de Silva et al., 2016; Williams et al., 2018), incur exorbitant treatment cost and exhaust antivenom stock which is inadequately supplied to begin with (Williams et al., 2011). The limited neutralization capacity of PCAV, nonetheless, is similar to that reported in a number of cobra venom neutralization studies, in which the *in vivo* neutralization effects of antivenom were consistently low (potency <1 mg/ml) (Tan et al., 2016b; Wong et al., 2016; Tan et al., 2017c; Tan et al., 2020; Wong et al., 2021). The key toxins (alpha-neurotoxins) in cobra venoms are small, less immunogenic proteins, and this presumably compromises the production of effective antibody and the neutralization activity of antivenom (Clementi et al., 1991). The development of more efficacious antivenom products for cobra bite neurotoxic envenomation is therefore warranted. This is achievable through improving the immunogen formulation with strategies such as protein-adjuvant conjugation and enrichment with toxins from multiple species known as “diverse toxin repertoire” (Bermúdez-Méndez et al., 2018; Ratanabanangkoon et al., 2020; Ratanabanangkoon, 2021).

## Conclusion

The present proteomic study unveiled a unique snake venom phenotype of *N. samarensis*, an Asiatic spitting cobra that represents the easternmost dispersal of *Naja* cobra species. Three-finger toxins (3FTx) constituted close to 90% of the total venom proteins, recording by far the highest 3FTx abundance in snake venom. The major 3FTx proteins in the venom were short-chain alpha-neurotoxins (SαNTX) which made up two-thirds of the total venom composition, while the long-chain alpha-neurotoxin (LαNTX) was absent. The venom proteomes of *N. samarensis* (current work) and *N. philippinensis* (Tan et al., 2019b) demonstrated that SαNTX are exclusively the neurotoxic components accountable for the venom lethality of these cobra species of high medical importance in Asia. Also, the abundance of cytotoxins (or cardiotoxins, and homologs) was considerably low (∼16%) in *N. samarensis* venom proteome compared to most other cobra species, and this is consistent with the lack of local tissue effect in envenomation caused by the Philippine cobras. Besides, the PLA_2_ content in the venom was exceptionally low (∼4%) for a spitting cobra, showing that the Philippine spitting cobras do not conform to the upregulating trend of PLA_2_ and CTX despite evolving the venom-spitting (spraying) trait. The hetero-specific antivenom PCAV was immunoreactive toward the *N. samarensis* venom and its toxin fractions including SαNTX, and the antivenom weakly cross-neutralized the toxin lethality albeit with a low potency. Together, the present study provides a deep insight into the venom composition and the pathophysiology of envenomation of this lesser-known spitting cobra species in Southeast Asia. The study also underscores the need for an antivenom product with improved efficacy in the region, which may be achieved by immunogen re-formulation to increase the neutralizing strength of the antibody against the principal neurotoxins as identified through venom proteomics and toxin-specific neutralization test.

## Data Availability

The datasets presented in this study can be found in online repositories. The names of the repository/repositories and accession number(s) can be found below: http://www.proteomexchange.org/, accession ID: PXD026270.
